# Soluble endoglin regulates expression of angiogenesis-related proteins and induction of arteriovenous malformations in a mouse model of hereditary hemorrhagic telangiectasia

**DOI:** 10.1242/dmm.034397

**Published:** 2018-09-21

**Authors:** Eunate Gallardo-Vara, Simon Tual-Chalot, Luisa M. Botella, Helen M. Arthur, Carmelo Bernabeu

**Affiliations:** 1 Centro de Investigaciones Biológicas, Consejo Superior de Investigaciones Científicas (CSIC), and Centro de Investigación Biomédica en Red de Enfermedades Raras (CIBERER), 28040 Madrid, Spain; 2 Institute of Genetic Medicine, Centre for Life, Newcastle University, Newcastle NE1 3BZ, UK

**Keywords:** Angiogenesis, Endoglin, HHT, AVM, TGF-β, Endothelial cells

## Abstract

Endoglin is a transmembrane glycoprotein expressed in vascular endothelium that plays a key role in angiogenesis. Mutations in the endoglin gene (*ENG*) cause hereditary hemorrhagic telangiectasia type 1 (HHT1), characterized by arteriovenous malformations (AVMs) in different organs. These vascular lesions derive from abnormal processes of angiogenesis, whereby aberrant vascular remodeling leads to focal loss of capillaries. Current treatments for HHT1 include antiangiogenic therapies. Interestingly, a circulating form of endoglin (also known as soluble endoglin, sEng), proteolytically released from the membrane-bound protein and displaying antiangiogenic activity, has been described in several endothelial-related pathological conditions. Using human and mouse endothelial cells, we find that sEng downregulates several pro-angiogenic and pro-migratory proteins involved in angiogenesis. However, this effect is much reduced in endothelial cells that lack endogenous transmembrane endoglin, suggesting that the antiangiogenic activity of sEng is dependent on the presence of endogenous transmembrane endoglin protein. In fact, sEng partially restores the phenotype of endoglin-silenced endothelial cells to that of normal endothelial cells. Moreover, using an established neonatal retinal model of HHT1 with depleted endoglin in the vascular endothelium, sEng treatment decreases the number of AVMs and has a normalizing effect on the vascular phenotype with respect to vessel branching, vascular density and migration of the vascular plexus towards the retinal periphery. Taken together, these data show that circulating sEng can influence vascular development and AVMs by modulating angiogenesis, and that its effect on endothelial cells depends on the expression of endogenous endoglin.

This article has an associated First Person interview with the first author of the paper.

## Abstract (precis)

**Summary:** Soluble endoglin regulates vascular development and arteriovenous malformations by modulating angiogenesis, and its effect on endothelial cells depends on expression of endogenous membrane-bound endoglin.

## INTRODUCTION

Endoglin is a homodimeric transmembrane glycoprotein that acts as an auxiliary receptor for members of the transforming growth factor-β (TGF-β) family of cytokines. It is expressed primarily in vascular endothelium, but also in mesenchymal cells, and plays a key role in vascular physiology, including angiogenesis and vascular remodeling (ten Dijke and Arthur, 2007; López-Novoa and Bernabeu, 2010; Núñez-Gómez et al., 2017; Redgrave et al., 2017; Ruiz-Llorente et al., 2017). In humans, there are two different protein isoforms, the predominantly expressed L-endoglin, and the minor isoform S-endoglin, generated by alternative splicing (Gougos and Letarte, 1990; Bellón et al., 1993; Blanco and Bernabeu, 2011). Both endoglin isoforms are identical in their extracellular and transmembrane regions, but they differ from each other in their cytoplasmic domain (Bellón et al., 1993). In addition to these two membrane-bound proteins, a circulating form of endoglin, originally named as soluble endoglin (sEng) (Venkatesha et al., 2006; Gregory et al., 2014), containing the extracellular region has been described. Circulating sEng is shed from membrane-bound endoglin by the proteolytic activity of the matrix metalloprotease 14 (MMP14 or MT1) (Hawinkels et al., 2010; Valbuena-Díez et al., 2012) and can be released from the placenta in exosomes enriched with certain sphingomyelin species, upon clustering with MMP14 (Ermini et al., 2017). However, the specific nature of the circulating sEng, whether as an individual soluble protein or complexed within exosomes, is still not fully understood. Shedding of sEng can be triggered by inflammation, tumor necrosis factor-α (TNF-α), endothelial injury or anti-endoglin antibodies (Li et al., 2003; Sunderland et al., 2011; Kumar et al., 2014; Gallardo-Vara et al., 2016). High levels of sEng have been reported in several endothelium-related pathological conditions (Bernabeu et al., 2009; Blázquez-Medela et al., 2010; Oujo et al., 2013; Gregory et al., 2014). Among these, markedly elevated levels of sEng are found in pre-eclampsia, a disease of high incidence in pregnant women which, if left untreated, can lead to the death of mother and baby (Venkatesha et al., 2006). Pre-eclampsia is characterized by hypertension and proteinuria associated with endothelial dysfunction. Several lines of evidence support a pathogenic role of sEng in pre-eclampsia, including antiangiogenic activity, increased vascular permeability and hypertension (Venkatesha et al., 2006; Hawinkels et al., 2010; Valbuena-Díez et al., 2012). Also, sEng has pro-inflammatory activity via nuclear factor kappa-light-chain-enhancer of activated B cells (NFκB) and interleukin-6 (IL6) (Varejckova et al., 2017), and can modulate inflammation-associated leukocyte adhesion and transmigration (Rossi et al., 2013). Studies in transgenic animals overexpressing human sEng suggest that sEng also contributes to endothelial dysfunction (Jezkova et al., 2016; Rathouska et al., 2017). Despite its critical importance in vascular pathology, the molecular mechanism of action of sEng remains poorly understood. It has been postulated that sEng activity is based on its capacity to antagonize the function of membrane-bound endoglin. For example, sEng binds to bone morphogenetic protein 9 (BMP9 or GDF2), a member of the TGF-β family, with high affinity (Castonguay et al., 2011; Alt et al., 2012; Saito et al., 2017) and this can lead to sequestration of BMP9, preventing its binding to surface endoglin and the subsequent downstream intracellular signaling of the TGF-β receptor complex (Venkatesha et al., 2006; Hawinkels et al., 2010; Gregory et al., 2014). Also, both membrane-bound endoglin and sEng contain an accessible arginine-glycine-aspartic acid (RGD) sequence, which is a consensus binding motif for integrin recognition (Gougos and Letarte, 1990; Saito et al., 2017), and it has been shown that sEng can inhibit integrin-mediated cell adhesion to endothelial endoglin, likely by competing with its binding to integrins (Rossi et al., 2013, 2016). Nevertheless, the exact molecular mechanisms of action of sEng remain to be elucidated.

Endoglin plays a key role in endothelial cells (ECs), as shown by numerous *in vitro* and *in vivo* studies. Roles include regulating cell proliferation and migration, actin cytoskeleton, endothelial nitric oxide synthase (eNOS) expression and activity, endothelial cell permeability, leukocyte extravasation, vessel maturation, arterial and venous specification, and vessel diameter in response to flow (López-Novoa and Bernabeu, 2010; Núñez-Gómez et al., 2017; Rossi et al., 2013, 2016; Mahmoud et al., 2010; Jerkic and Letarte, 2015; Baeyens et al., 2016; Sugden et al., 2017; Jin et al., 2017). In addition, mutations in the human endoglin gene (*ENG*) are the underlying cause of hereditary hemorrhagic telangiectasia (HHT) type 1 (HHT1), an autosomal-dominant disorder characterized by the presence of arteriovenous malformations (AVMs) in different organs (McAllister et al., 1994; Abdalla and Letarte, 2006; Shovlin, 2010). It has been postulated that the vascular lesions derive from abnormal processes of angiogenesis and vascular remodeling, leading to focal loss of capillaries and, as a consequence, a direct connection between venules and arterioles (Wetzel-Strong et al., 2017; Cunha et al., 2017). The current treatments for HHT1 include several antiangiogenic therapies (Ruiz-Llorente et al., 2017; Ardelean and Letarte, 2015). Interestingly, sEng displays antiangiogenic activity (Hawinkels et al., 2010; Venkatesha et al., 2006), but its putative role in AVM formation and treatment has not yet been explored.

In this study, we find that sEng downregulates several pro-angiogenic and pro-migratory proteins involved in angiogenesis, and this effect is dependent on the presence of endogenous transmembrane endoglin. Furthermore, using an inducible endoglin (*Eng*) knockout (KO) animal model, we show that sEng treatment decreases the number of AVMs in the neovascularized retina. Taken together, this work reveals, for the first time, the context-dependent role of sEng in regulating angiogenesis and vascular pathogenesis.

## RESULTS

### sEng inhibits endothelial tubulogenesis and cell migration

**Fig. 1.**
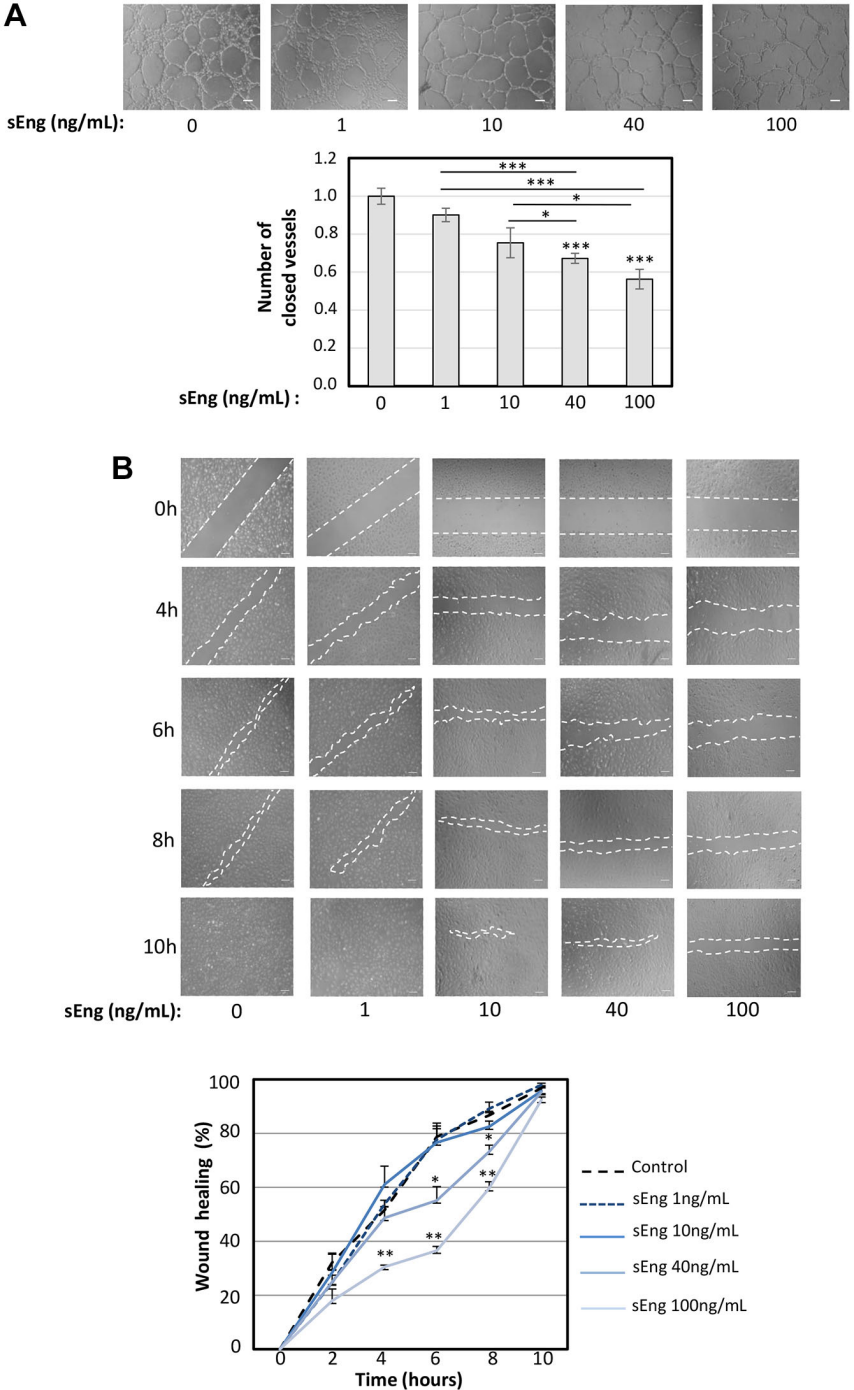
**Effect of sEng on tubulogenesis and wound healing.** (A) HUVECs were incubated on Matrigel plates at 37°C in VEGF-enriched EBM2/EGM2 medium containing increasing doses of sEng (0-100 ng/ml). The cord network formation was visualized by taking pictures at different times up to 6 h after cell plating. The appearance of a complete network is achieved by 6 h in untreated cells or cells treated with 1 ng/ml sEng, while at higher concentrations of sEng, cells remain in open tubules with some patches of disorganized and sparse cells. A representative assay of more than three different experiments per condition is shown. Scale bars: 100 µm. The tube density (closed tubes) in the network was quantified, normalized and represented in the histogram. *P<0.05; ***P<0.001. (B) Confluent HUVECs monolayers were disrupted with a pipette tip and treated with increasing doses of sEng. Photos were taken at different times and the speed of migration was quantified by densitometry of the filled space (area within white dashed lines) in the wound at each time point (bottom graph). The speed of migration to close the wound of sEng-treated cells is slower than that of untreated ones. Experiments were repeated three times and a representative photograph of each condition is shown. Scale bars: 100 µm. *P<0.05; **P<0.01.

The effect of sEng on tubulogenesis and in wound healing assays was analyzed in cultured human umbilical vein-derived endothelial cells (HUVECs) using a physiological range of sEng concentrations: low (1-10 ng/ml) and mid to high concentrations (40-100 ng/ml). For 3D tubulogenesis assays, HUVECs were cultured on vascular endothelial growth factor (VEGF)-containing Matrigel, and treated with increasing concentrations of sEng (Fig. 1A). After 6 h of culture with sEng, a dose-dependent inhibition of tubular network formation was observed, compared with control HUVECs without sEng treatment. This inhibition was most evident at doses of 40 ng/ml and 100 ng/ml sEng. Next, cell migration experiments were performed by measuring the endothelial ‘scratch wound’ closure of HUVECs monolayers over time (Fig. 1B). Under normal conditions, without sEng treatment, the wound was almost closed between 6 h and 8 h (75% and 90%, respectively). However, at 100 ng/ml sEng, closure was only 35% at 6 h and 60% at 8 h (Fig. 1B), suggesting that sEng induced an inhibitory effect. Because this type of wound healing assay measures a combination of cell migration and proliferation, we also analyzed the individual effect of sEng on HUVEC proliferation. We found no effect of sEng on cell proliferation (data not shown), suggesting that the overall changes observed in the scratch wound assay are mainly due to inhibition of cell migration by sEng. Our observations that sEng inhibits both endothelial cell tubulogenesis and migration are in agreement with the reported antiangiogenic activity *in vivo* of sEng, including the inhibition of VEGF-induced blood vessel formation and sprouting (Venkatesha et al., 2006; Hawinkels et al., 2010; Castonguay et al., 2011).

### sEng affects the expression of soluble proteins related to angiogenesis in HUVECs and MLECs

**Fig. 2.**
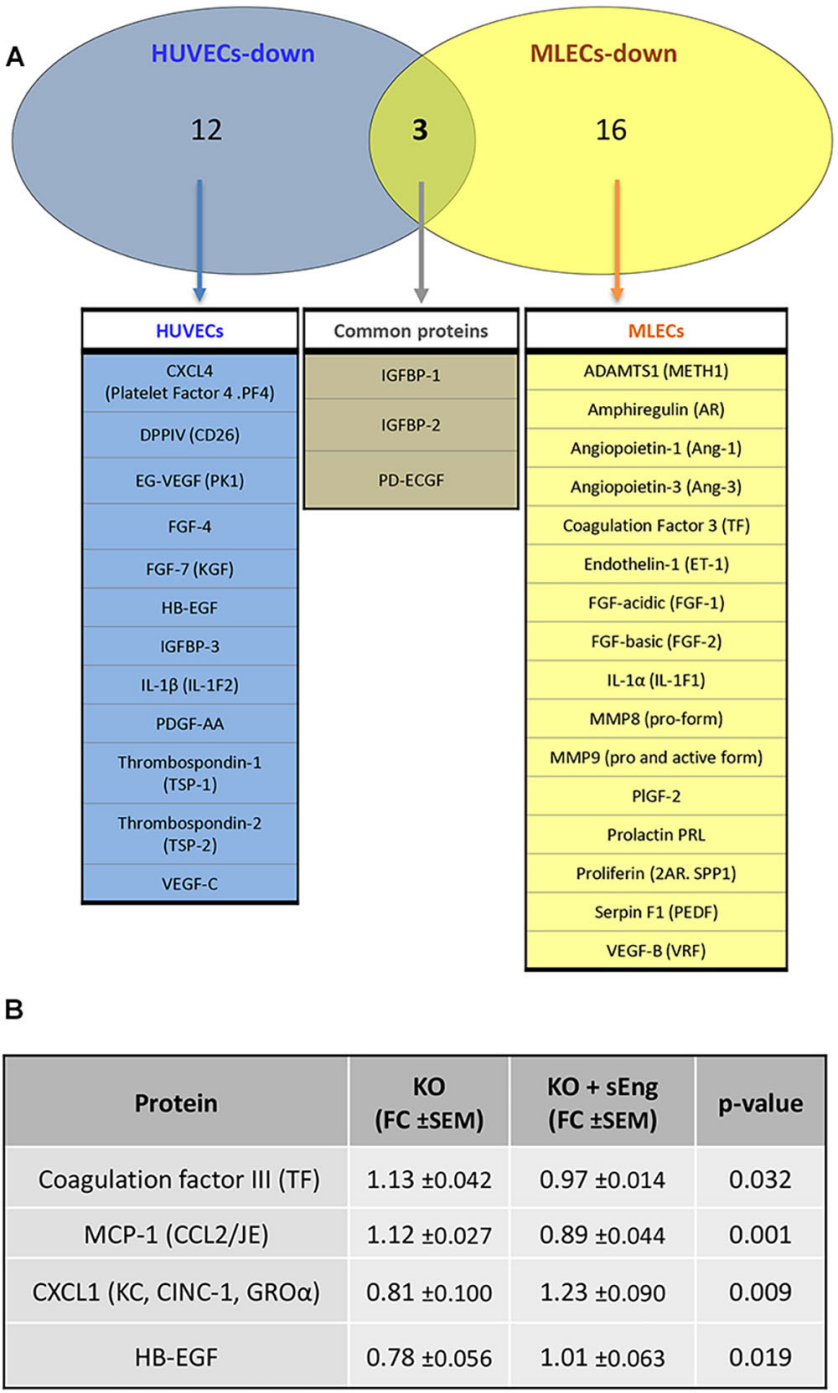
**Analysis of deregulated proteins upon treatment with sEng.** Differentially expressed secreted angiogenic proteins in HUVECs, MLECs and *Eng*-KO MLECs, previously identified in protein arrays, using a cutoff of 0.9 or 1.1 (Tables S1 and S2) were analyzed. (A) Venn diagram representing the differential protein expression between HUVECs and MLECs upon treatment with sEng. Among the downregulated proteins, from human and mouse panels, 12 were found only in HUVECs, 16 were found only in MLECs and three were found in both HUVECs and MLECs. Within this last set of proteins, statistical significance in both cell types was found for IGFBP-1, IGFBP-2 and PD-ECGF. (B) ELISA analysis of differentially secreted angiogenic proteins in *Eng*-KO MLECs in the absence (KO) or presence (KO+sEng) of soluble endoglin compared with control MLECs. Results were normalized to the total protein concentration in supernatants, and compared with untreated MLECs, which was given an arbitrary value of 1. For each protein, a minimum of four different experiments, each in triplicate, were carried out. Fold change (FC) measurements±s.e.m. and *P*-values between sEng-treated and untreated KO MLECs are represented.

To investigate possible molecular mechanisms underlying the antiangiogenic activity of sEng, conditioned media from HUVECs and mouse lung endothelial cells (MLECs) were analyzed following treatment with sEng. Changes in the secreted angiogenic protein profile were assessed using angiogenesis-specific antibody arrays. Upon treatment with sEng, HUVECs and MLECs reduced the expression of 15 of 55 (HUVECs), and 19 of 53 (MLECs), angiogenic proteins present in both human and mouse arrays (Table S1A). Most of the downregulated proteins have pro-angiogenic properties, consistent with the antiangiogenic and antimigratory effects of sEng observed above. Ascertaining which angiogenesis-related proteins were downregulated in both cell types was partly limited by the fact that the arrays had only 72% proteins common to both human and mouse panels. Nevertheless, sEng treatment led to significant downregulation of three pro-angiogenic proteins in both human and mouse ECs [insulin-like growth factor-binding protein 1 (IGFBP-1) and 2 (IGFBP-2), and platelet-derived endothelial cell growth factor (PD-ECGF)] (Fig. 2A; Table S1A). An additional six proteins [endothelin, CXCL-4 (PF4), dipeptidyl peptidase 4 (DPP4), heparin binding-like epidermal growth factor (HB-EGF), platelet-derived growth factor (PDGF-AA or PDGFA) and thrombospondin-2 (TSP-2 or THBS2)] appeared to be reduced in both human and mouse ECs, but reached statistical significance in only one cell type (Fig. 2A; Table S1A). In contrast to the striking decrease in pro-angiogenic proteins following sEng treatment, only two proteins in HUVECs and none in MLECs were significantly increased in response to sEng (Table S1B). Interestingly, maspin, one of the upregulated proteins, displayed angioinhibitory activity, in line with an antiangiogenic effect of sEng treatment.

### sEng regulates the secretion of angiogenic proteins and this effect is dependent on endoglin gene expression

To determine whether sEng treatment had a similar antiangiogenic effect on endoglin-deficient cells, as a model of HHT1, we used a MLEC line isolated from *Eng^fl/fl^;RosaCre^ERT^* mice (Anderberg et al., 2013). The efficiency of 4-OH-tamoxifen-induced endoglin gene silencing in these MLECs was confirmed by RT-PCR and immunostaining (Fig. S1). Differentially expressed angiogenic proteins from *Eng*-KO and control MLECs, with and without treatment with sEng, were determined using the mouse angiogenic protein array (Table S2). *Eng*-KO MLECs show altered expression of many angiogenic proteins compared with controls, in line with the known importance of endoglin in angiogenesis. However, sEng treatment normalizes several of these differences back to control levels. Of eight angiogenic proteins significantly downregulated in *Eng*-KO MLECs (including endogenous Eng), one was restored to normal values upon treatment with sEng, whereas there was a similar trend for several other proteins (Table S2). These include heparin-binding EGF-like growth factor (HB-EGF), involved in vascular remodeling; the chemokine (C-X-C motif) ligand 1 (CXCL1), involved in angiogenesis, arteriogenesis, inflammation, and wound healing; pentraxin-3 (PTX3), an acute-phase-response protein that regulates angiogenesis after ischemia; and proliferin, a prolactin/growth hormone-like peptide with angiogenic properties. In addition, expression of angiogenin, a ribonuclease with potent proangiogenic activity, and placental growth factor (PlGF or PGF), a VEGF family member involved in angiogenesis and vasculogenesis, was increased towards normal levels following sEng treatment. Similarly, of three proteins significantly upregulated in *Eng-*KO MLECs, two were reduced to normal levels by sEng. These were coagulation factor III, also known as tissue factor (TF), involved in the initial steps of blood coagulation, and PD-ECGF, a thymidine phosphorylase which promotes angiogenesis *in vivo* and stimulates the growth of ECs *in vitro*. Several other upregulated proteins, including the pro-inflammatory chemokine MCP-1 (CCL2), showed a similar trend of restoration towards normal by sEng treatment (Table S2). To confirm this trend, enzyme-linked immunosorbent assay (ELISA) was used to ascertain the levels of two upregulated (coagulation factor III and MCP-1) and two downregulated (HB-EGF and CXCL1) proteins in *Eng-*KO MLECs with and without sEng treatment (Fig. 2B). This analysis confirmed the array data showing that for all four proteins, sEng significantly altered their levels towards that of controls. Taken together, these results show that sEng can partially rescue the angiogenic protein expression imbalance observed in *Eng*-KO MLECs, suggesting that sEng can help to compensate for the lack of endogenous transmembrane endoglin.

### sEng inhibits the development of retinal arteriovenous malformations in an HHT1 murine model

**Fig. 3.**
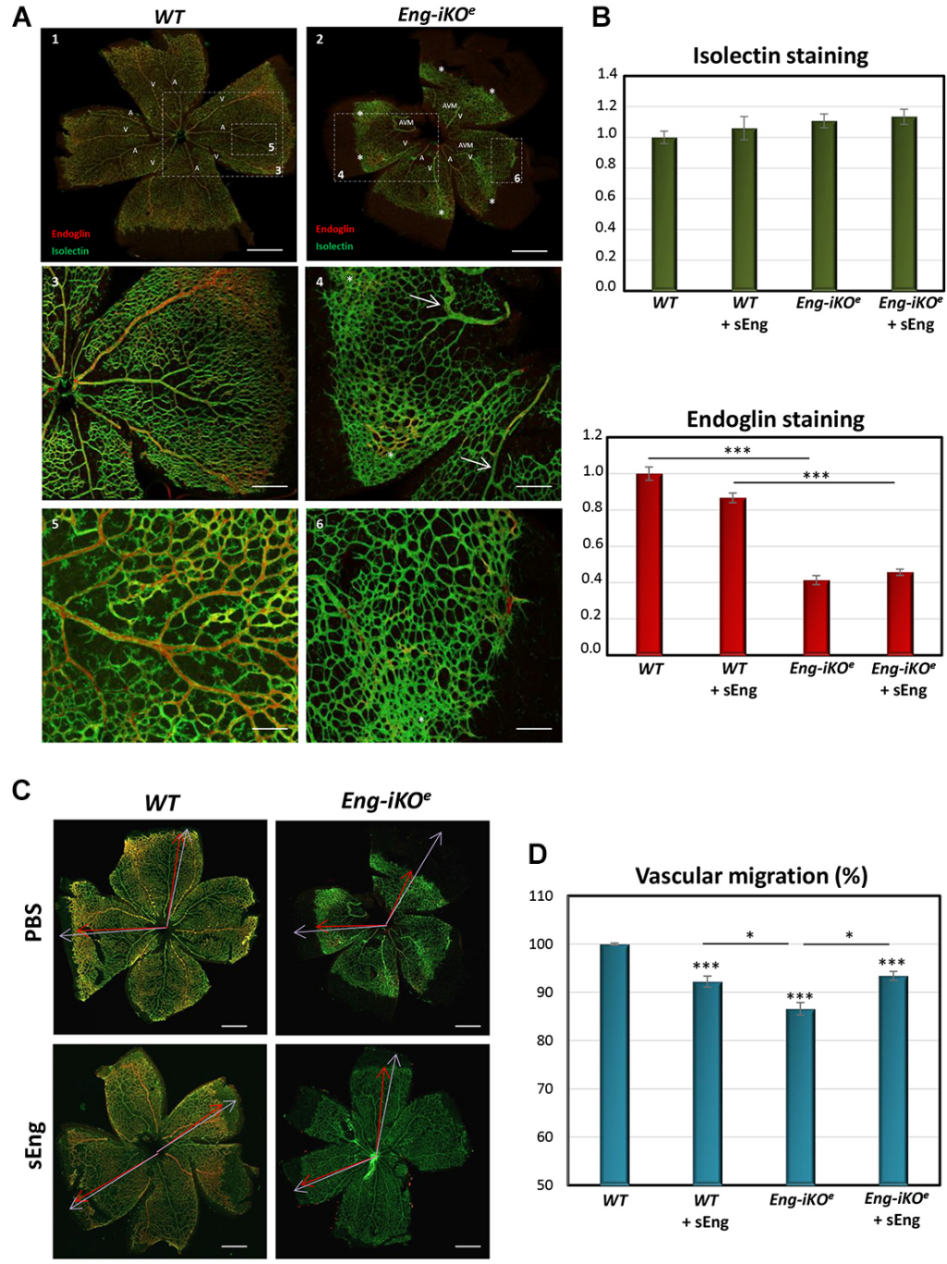
**Analysis of vascular markers and migration in retinas.** (A) P7 retinas from *Eng*-iKO^e^ and WT mice were stained with isolectin (green) and anti-endoglin (red), visualized by fluorescence microscopy and analyzed using the Fiji-ImageJ program. Decreased endothelial endoglin expression and increased abnormalities in the neonatal vascular plexus from retinas of *Eng*-iKO^e^ mice compared with WT animals were observed. The presence of veins (V) and arteries (A) is indicated. Endoglin staining (red fluorescence) predominates in veins. Loss of endoglin protein expression in endothelial cells leads to AVMs (arrows) and hyperbranching in the periphery of retinas (asterisk). White dotted line boxes in upper panels represent regions enlarged in lower panels. Images are taken at different magnifications (5×, 10× or 20×). Scale bars: 500 µm (1,2), 400 µm (3,4) and 100 µm (5,6). (B) Quantification of vascular staining in sEng-intraocularly treated and untreated mice. WT and *Eng*-iKO^e^ mice were treated with or without sEng, as indicated. Fluorescence intensity of each marker was quantified using the Fiji-ImageJ2 software, and normalized with respect to WT controls. Endoglin expression is markedly decreased in *Eng*-iKO^e^ versus WT retinas, whereas it is not affected by sEng treatment. (***P<0.001). *n*>20 mice per condition. (C,D) Analysis of vascular migration. (C) Examples of stained retinas to illustrate the measurement of vascular and retina radius are shown. Red arrows indicate the vascular radius (measurement from the center to the edge of the vascular front), whereas purple arrows indicate the retina radius (measurement from the center to the edge of retina). The black areas indicate areas in which there has been some breakage in the fragile retina. Magnification, 5×. Scale bars: 500 µM (D) Quantification of vascular migration. The vascular radius/retina radius ratio, represented as a percentage, with respect to the control, was used to quantify vascular migration. Progression of the vascular plexus to retinal periphery is delayed in *Eng*-iKO^e^ compared with WT control retinas, and this delay is partly reversed when *Eng*-iKO^e^ animals are treated with sEng. In WT retinas, sEng treatment decreases vascular migration. ***P<0.001 compared with the WT condition; *P<0.05 between the indicated conditions. *n*=30 (WT), *n*=22 (WT+sEng), *n*=27 (*Eng*-iKO^e^) and *n*=24 (*Eng*-iKO^e^+sEng).

**Fig. 4.**
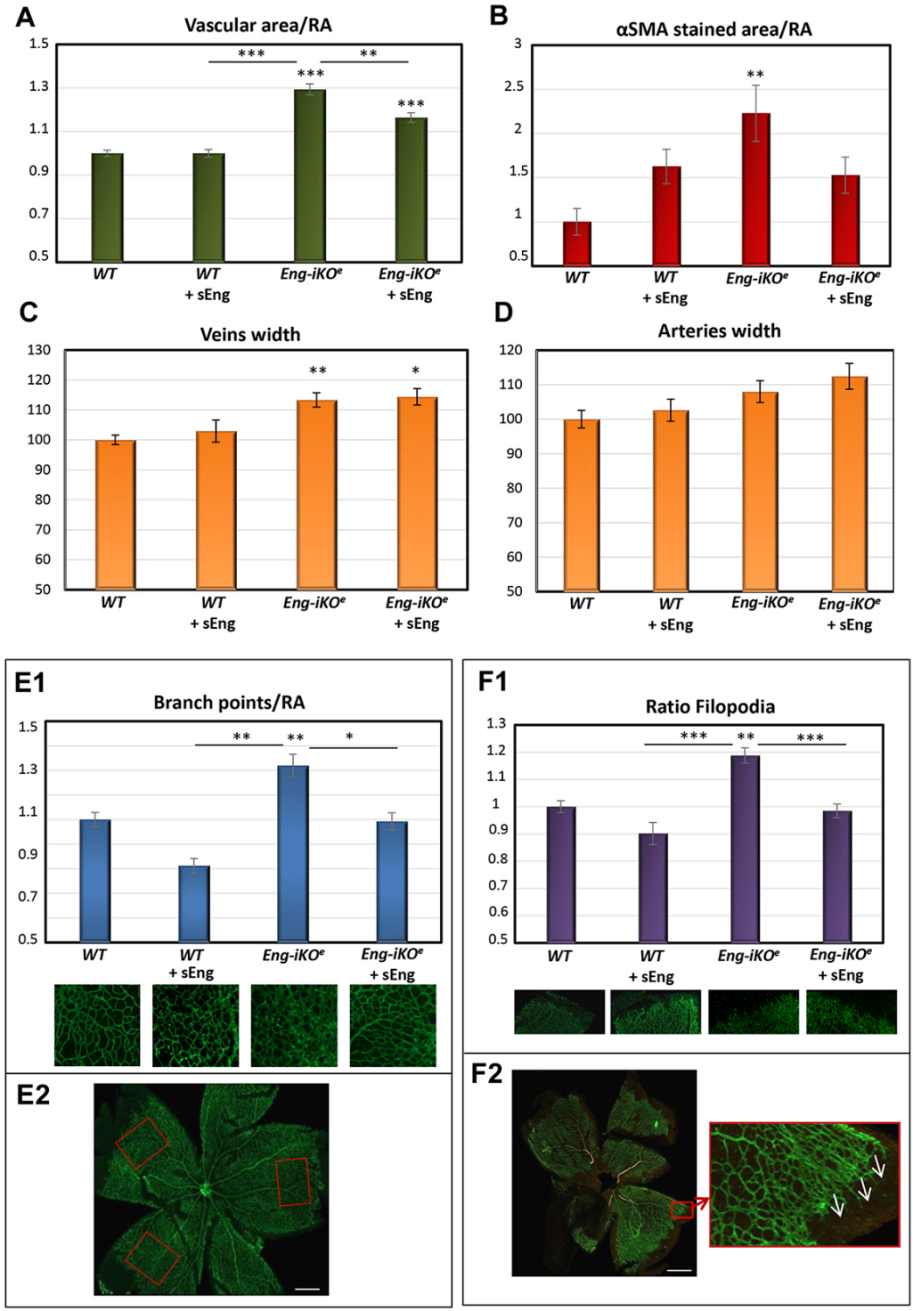
**Effect of sEng on vascular parameters of the retinal plexus from *Eng*-iKO^e^ mice.** WT and *Eng*-iKO^e^ mice were treated with or without sEng, as indicated. Retinas were stained with isolectin, anti-endoglin or anti-αSMA, and the vasculature was visualized by fluorescence microscopy (Fig. S2). Fluorescence intensity, as well as the area covered by each marker, were quantified using the Fiji-ImageJ2 software, and normalized with respect to WT controls. (A) Vascular density was measured in P7 neonatal retinas using isolectin-stained area and normalized to the corresponding retinal area (RA). *n*=34 (WT), *n*=27 (WT+sEng), *n*=31 (*Eng*-iKO^e^) and *n*=17 (*Eng*-iKO^e^+sEng) per condition. As a reference, the proportion of the retina covered by the vascular plexus, as measured by isolectin staining (vascular area/RA) is 35±2.5% in the WT. (B) αSMA-stained area normalized to RA. *n*=14 (WT), *n*=10 (WT+sEng), *n*=13 (*Eng*-iKO^e^) and *n*=13 (*Eng*-iKO^e^+sEng). (C,D) Cross-sectional width or caliber of veins (C) and arteries (D) were measured in at least three different vessels of each retina, and in three different areas of each vessel at a similar distance from the center of the retina. *n*=40 (WT), *n*=20 (WT+sEng), *n*=26 (*Eng*-iKO^e^) and *n*=21 (*Eng*-iKO^e^+sEng) per condition. (E1) Branch points normalized to the corresponding RA. *n*=36 (WT), *n*=17 (WT+sEng), *n*=24 (*Eng*-iKO^e^) and *n*=15 (*Eng*-iKO^e^+sEng). (F1) The number of filopodia, leading edge of migrating tip cells, was normalized to the selected field of view. *n*=39 (WT), *n*=20 (WT+sEng), *n*=25 (*Eng*-iKO^e^) and *n*=23 (*Eng*-iKO^e^ +sEng) per condition. Representative images made with digital zoom are shown under each condition in E1 and F1. E2 and F2 illustrate examples of the RAs (red outline boxes) in which branch points/capillary junctions (E2) and filopodia on sprouting cells in the periphery (F2) were measured. Scale bars: 500 µm. Vessel density (A), specific area covered by αSMA staining (B), vessel branch points or capillary unions (E1) and number of sprouts (F1) are all significantly increased in *Eng*-iKO^e^ retinas, compared with controls (WT). However, sEng treatment is able to restore these abnormal parameters, yielding a phenotype similar to that of WT retinas. (*P<0.05; **P<0.01; ***P<0.001; compared with WT, unless indicated between specified conditions).

We noticed that many of the genes encoding those proteins identified in our study (Fig. 2B) are expressed in endothelial cells during development of the mouse retina (Jeong et al., 2017), which is a widely used animal model of angiogenesis (Selvam et al., 2018). In order to assess the effect of sEng on AVM formation during vascular development, we turned to endothelial-specific tamoxifen-inducible endoglin KO (*Eng*-iKO^e^) mice, which develop AVMs in the neonatal retina (Mahmoud et al., 2010). Wild-type (WT) and endothelial-specific *Eng*-iKO^e^ mice were each intraocularly injected with sEng in the left eye and vehicle (PBS) in the right eye. Retinal vasculature was visualized 2 days later by immunofluorescent staining. At the retinal periphery, where there is active proliferation of ECs involved in angiogenesis, endoglin expression was increased compared with the remodeled vessels of the central zone (Fig. 3A; Fig. S2A). Whereas isolectin staining did not show any significant difference among the four conditions, endoglin staining was decreased in *Eng*-iKO^e^ retinas, compared with WT samples, as expected (Fig. 3B). We confirmed that sEng treatment did not affect the level of endoglin staining, when comparing treated versus untreated mice, thus ruling out a potential artifact owing to sEng protein accumulation in the vasculature. As previously described (Mahmoud et al., 2010), loss of endoglin expression in ECs led to the formation of AVMs within the retinal plexus, delayed progression of the vascular plexus towards the periphery, and hyperbranching of the peripheral vessels (Fig. 3). The effect of sEng treatment on these vascular parameters in WT and *Eng*-iKO^e^ retinas was assessed. We show here, for the first time, that treatment with sEng inhibits vascular plexus migration in WT retinas (Fig. 3C,D), a finding compatible with the antimigratory and antiangiogenic activity of sEng observed *in vitro* (Fig. 1). In contrast, sEng treatment of *Eng*-iKO^e^ retinas favors vascular migration, suggesting a possible mechanism of phenotype ‘normalization’ or ‘rescue’ by sEng. Normalized vascular density and area covered by alpha smooth muscle actin (αSMA) staining were higher in *Eng*-iKO^e^ (>30%) compared with WT retinas (Fig. 4A,B), and this phenotype was also partially rescued by sEng treatment, which decreased vascular density by ∼15%, but had no significant effect on vascular density of WT retinas (Fig. 4A). In addition, a significant increase in caliber or width of veins, but not of arteries, in *Eng*-iKO^e^ retinas, compared with WT retinas was observed, although this was not affected by sEng treatment (Fig. 4C,D).

Compared with WT retinas, *Eng*-iKO^e^ retinas display more capillary junctions or branching (Fig. 4E1,E2) in the peripheral areas, where angiogenesis is more active, and a higher number of filopodia at the leading edge of migrating tip cells (Fig. 4F1,F2). However, intraocular treatment with sEng significantly decreased both branching and filopodia in *Eng*-iKO^e^ retinas (Fig. 4E1,F1) by 19% (branching) and 17% (filopodia number), thus partially restoring the phenotype of *Eng*-iKO^e^ retinas to that of WT.

**Fig. 5.**
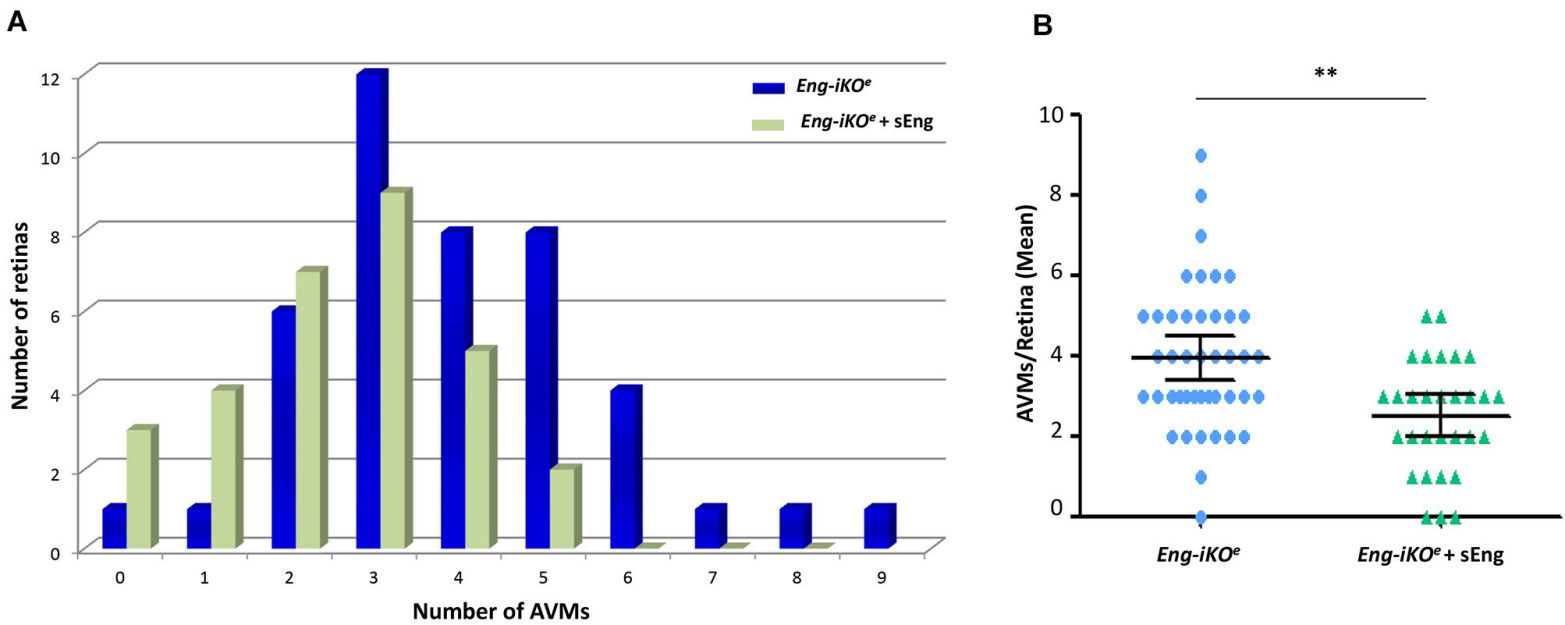
**Effect of sEng treatment on the number of AVMs in *Eng*-iKO^e^ retinas.** AVMs from at least 30 retinas from each group were analyzed. (A) Histogram representation of the number of AVMs found relative to the number of counted retinas. Retinas treated with sEng show a lower incidence of AVMs than untreated retinas. (B) Mean number of AVMs corrected for the number of retinas analyzed in *Eng*-iKO^e^ mice, treated with or without sEng. Values show 95% confidence intervals. **P<0.01. *n*=43 (*Eng*-iKO^e^) and *n*=30 (*Eng*-iKO^e^+sEng) retinas per condition.

Based on the observed role of sEng on angiogenesis and vascular remodeling, we next examined the effect of sEng on AVM formation in *Eng*-iKO^e^ retinas (Fig. S2A,B). Treatment with sEng showed a tendency to decrease the size of AVMs (Fig. S3A,B). Moreover, sEng significantly decreased the incidence of AVMs in *Eng*-iKO^e^ retinas when compared with vehicle treatment (Fig. 5A; Fig. S2A,B). Overall, the mean number of AVMs was reduced from 4.0 to 2.5 AVMs/retina in *Eng*-iKO^e^ mice upon treatment with sEng (Fig. 5B).

In the neonatal retina, vascular smooth muscle cell (vSMC) coverage is associated with muscularized arteries, whereas it is normally absent from veins at this stage of development. We find that the development of AVMs is associated with an ‘arteriolization’ effect owing to increased blood flow through these vessels, leading to an increased number of αSMA-positive vSMCs at the site of AVMs (Fig. S2B), as reported (Mahmoud et al., 2010). Of note, we found a significantly increased expression of αSMA in *Eng*-iKO^e^ retinas (compared with WT retinas) that was associated with AVMs (Fig. 4B; Figs S2B and S3C). Importantly, the area occupied by vSMCs in *Eng*-iKO^e^ retinas is significantly decreased after sEng treatment (Fig. 4B; Fig. S3C), consistent with the reduced caliber, area (Fig. S3A,B) and number (Fig. 5) of AVMs.

Taken together, the above results (summarized in Table 1) show that sEng can partially compensate for membrane-bound endoglin insufficiency to promote rescue of vascular defects in *Eng*-iKO^e^ mice.

**Table 1.**
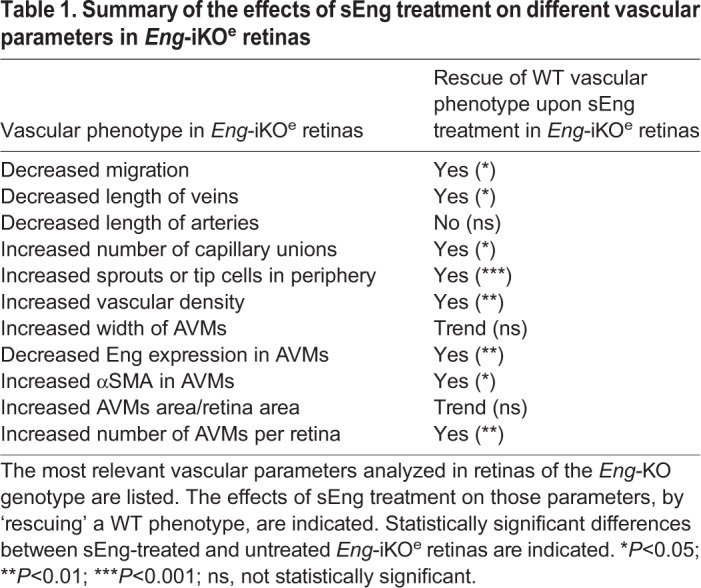
**Summary of the effects of sEng treatment on different vascular parameters in *Eng*-iKO^e^ retinas**

## DISCUSSION

Abnormal levels of sEng are found in several endothelium-related pathological conditions, including pre-eclampsia (Venkatesha et al., 2006; Oujo et al., 2013; Gregory et al., 2014), atherosclerosis, hypercholesterolemia (Blann et al., 1996; Blaha et al., 2008; Rathouska et al., 2015), diabetes mellitus (Blázquez-Medela et al., 2010; Ceriello et al., 2015; Emeksiz et al., 2016), hypertension (Blázquez-Medela et al., 2010), diabetic retinopathy (Malik et al., 2005), coronary artery disease (Li et al., 2000a; Ikemoto et al., 2012; Saita et al., 2017), HHT1 (Letarte et al., 2005; Botella et al., 2015), acute myocardial infarction (Cruz-Gonzalez et al., 2008) and cancer (Li et al., 2000b; Bernabeu et al., 2009). In many of these diseases, the deregulated levels of sEng in plasma, serum or urine from patients have been postulated to be a reliable biomarker for severity correlation and prognosis. Furthermore, sEng appears to play an active role in disease pathogenesis. For example, it has been reported that, in pre-eclampsia, sEng contributes to hypertension and renal involvement (Venkatesha et al., 2006; Valbuena-Díez et al., 2012); in atherosclerosis, sEng induces a pro-inflammatory response, leading to endothelial dysfunction (Varejckova et al., 2017; Jezkova et al., 2016); and, in cancer, sEng acts as an antiangiogenic protein by inhibiting the ongoing neoangiogenesis associated with the growth of solid tumors (Hawinkels et al., 2010; Castonguay et al., 2011). In spite of the wide range of pathophysiological effects of sEng reported in the cardiovascular system, its underlying mechanism of action on ECs is poorly understood. Of note, the balance between pro- and antiangiogenic factors in angiogenesis/vascular remodeling is crucial (Potente and Carmeliet, 2017). One of the aims of this work was to analyze the levels of angiogenesis-related proteins released from ECs in the presence of sEng, to provide some insights into the altered angiogenesis/vascular remodeling processes associated with increased circulating levels of endoglin. Using human and mouse ECs, both expressing high levels of membrane-bound endoglin, we found that sEng induced a protein expression pattern with a predominant antiangiogenic profile. After sEng treatment, the levels of secreted pro-angiogenic proteins, such as VEGF, fibroblast growth factor (FGF), PDGF, IGFBP proteins and thrombospondin, were all significantly decreased, consistent with the reported antiangiogenic effect of sEng. In this regard, sEng stimulated the expression of only one pro-angiogenic protein (leptin) and the antiangiogenic protein (maspin) (Table S1B). Surprisingly, the cellular response to sEng in the absence of membrane-bound endoglin yielded different response to ECs expressing normal levels of endoglin (Tables S1 and S2). Indeed, altered levels of secreted angiogenic proteins in *Eng*-iKO^e^ ECs, including coagulation factor III, PD-ECGF, HB-EGF, CXCL1 and MCP-1, tend to be normalized towards that of control ECs after treatment with sEng (Fig. 2B; Table S2B). These results clearly indicate that membrane-bound endoglin is key in the regulation of the angiogenic signaling and its absence produces an imbalance that can be compensated by sEng.

Because endoglin is a component of the TGF-β receptor complex (López-Novoa and Bernabeu, 2010; Mahmoud et al., 2011), it is tempting to speculate that the effects of sEng on protein expression are mediated by this signaling pathway. In this regard, it is worth mentioning that SERPINE1 (also known as PAI-1), a characteristic downstream target of the TGF-β route in ECs. Thus, basal levels of PAI-1 in *Eng*-iKO^e^ cells tend to be higher than those in control ECs (Table S2B), a finding compatible with the upregulated expression of the *PAI-1* gene found in gene arrays of ECs derived from HHT patients (Fernandez-Lopez et al., 2007) and with increased ALK5 (TGFBR1) signaling in the absence of endoglin (Lebrin et al., 2004). However, sEng treatment tends to restore PAI-1 levels in *Eng*-iKO^e^ cells to similar levels of control ECs (Table S2B), potentially by restoring the normal balance of the TGF-β signaling pathway. Of note, the extracellular part of endoglin specifically interacts with the TGF-β type I receptors ALK1 (ACVRL1) and ALK5 and with the TGF-β type II receptor (Guerrero-Esteo et al., 2002; Blanco et al., 2005). Also, endoglin is released from the placenta into the maternal circulation via sphingomyelin (18:0)-enriched exosomes, together with ALK5 and the TGF-β type II, where it can associate with these receptors, forming a functional receptor complex and modulating the vascular effects of TGF-β in the circulation (Ermini et al., 2017). Although soluble endoglin does not bind on its own to TGF-β1 (Gregory et al., 2014), it is possible that sEng, which contains most of the extracellular part of endoglin, could bind to the TGF-β receptor complex, mimicking membrane-bound endoglin and thus modulating its downstream signaling. This is an interesting possibility that could explain why in endoglin-silenced ECs, sEng restores the protein expression pattern of normal endothelial cells, and in an HHT1 animal model, sEng decreases the number of AVMs.

**Fig. 6.**
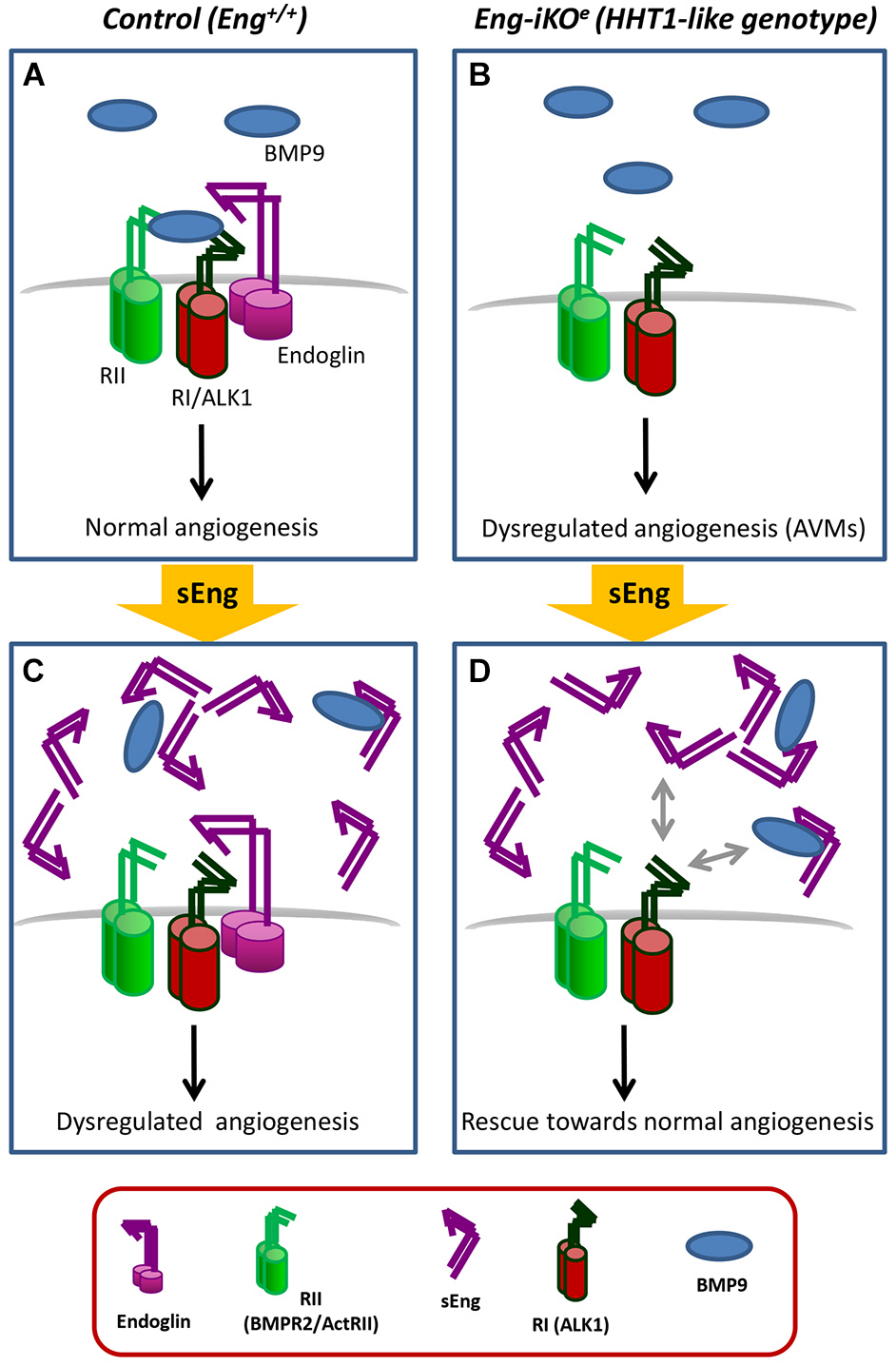
**Hypothetical model of sEng action on the vasculature.** (A,B) In normal endothelial cells, membrane endoglin is a component of a receptor complex that contains type I (RI; ALK1), and type II (RII; BMPR2/ActRII) TGF-β receptors, which can be activated by BMP9, leading to an equilibrium between pro- and antiangiogenic factors (A). In endoglin-silenced endothelial cells (*Eng*-iKO^e^), BMP9 cannot bind to endoglin and BMP9-dependent signaling is disturbed, leading to a dysregulated expression of angiogenic factors, decreased migration during angiogenesis and the presence of AVMs (B). (C,D) In normal endothelial cells, the circulating extracellular region of endoglin (sEng) can interact with BMP9, sequestering the ligand, interfering with the intracellular signaling of the receptor complex and changing the angiogenesis balance towards a dysregulated state (C). In *Eng*-iKO^e^, BMP9 cannot bind to membrane endoglin, but interacts with sEng, and the resulting BMP9/sEng complex interacts with the ALK1 receptor on the cell membrane, enhancing the proangiogenic ALK1 signaling and decreasing the incidence of AVMs (D). The involvement of endoglin in the TGF-β1/ALK5 signaling pathway of endothelial cells has been omitted for simplification.

The extracellular region of endoglin binds with high affinity to BMP9 (Castonguay et al., 2011; Alt et al., 2012; Saito et al., 2017), a TGF-β family member involved in the development of blood and lymphatic vessels (Levet et al., 2013; Chen et al., 2013; Li et al., 2016) and in endoglin-dependent chemokine responses of ECs (Young et al., 2012). In addition to HHT1 patients carrying mutations in endoglin, an HHT-like disorder has also been reported in patients heterozygous for mutations in BMP9 (Wooderchak-Donahue et al., 2013), whereas single-allele mutations in the *ALK1* gene give rise to HHT2 (Johnson et al., 1996). Accordingly, BMP9, ALK1 and endoglin proteins participate in a common signaling pathway also involving the type II receptors BMPRII and ActRII (Mahmoud et al., 2011; Tillet and Bailly, 2015; Roman and Hinck, 2017; Ruiz-Llorente et al., 2017) (Fig. 6A,B). Thus, in normal ECs it is possible that upon binding to BMP9, sEng (either alone or in complex with exosomes), ‘hijacks’ the ligand, prevents its binding to the receptor complex, and inhibits the downstream intracellular signaling (Hawinkels et al., 2010; Gregory et al., 2014; Venkatesha et al., 2006; Wang et al., 2017) (Fig. 6C). However, in endoglin-silenced ECs, BMP9 cannot bind to membrane endoglin, but can still associate with sEng (Castonguay et al., 2011; Saito et al., 2017). Furthermore, because sEng-bound BMP9 can directly interact with ALK1 (Blanco et al., 2005; Castonguay et al., 2011; Saito et al., 2017), it can be hypothesized that the effects of BMP9/sEng involve its interaction with ALK1 to promote proangiogenic ALK1 signaling and decrease the incidence of AVMs (Fig. 6D). As sEng and the type II receptors BMPRII or ActRII bind to BMP9 in a mutually exclusive fashion, sEng would be released, potentially enabling repeated enhancement of signaling. However, further detailed studies are needed to elucidate the exact mechanism of action of sEng in this pathway.

As discussed above, sEng displays antiangiogenic activity and the protein array data suggest that sEng regulates the expression of different proteins involved in angiogenesis and vascular remodeling, which are key processes involved in the generation of AVMs. In addition, using an HHT1 model of ECs, sEng was able to partly restore the protein expression pattern of normal ECs. Because several antiangiogenic therapies have been used in HHT patients (Lebrin et al., 2010; Dupuis-Girod et al., 2012, 2016; Albiñana et al., 2012) to inhibit vascular bleeding produced by the rupture of AVMs, we analyzed whether sEng could also modulate the occurrence of AVMs formed in an established mouse model of HHT1 (Mahmoud et al., 2010; Tual-Chalot et al., 2015). In the absence of endoglin, retinal vascular development shows delayed vascular plexus remodeling, increased endothelial cell proliferation, formation of AVMs, and increased SMA expression in AVMs as a result of increased flow (Mahmoud et al., 2010). We find that intraocularly injected sEng promotes rescue of abnormal vasculature, and decreases the incidence and size of AVMs in this model (Table 1). Upon sEng treatment, the number of AVMs was decreased, the number of capillary unions and sprouts of tip cells in the periphery were normalized, and the vascular density and the length of veins were restored, with respect to the WT phenotype.

It has been proposed that sEng might play a role in promoting AVMs (Chen et al., 2009). Patients with sporadic brain AVMs had higher levels of sEng compared with controls, but membrane-bound endoglin levels were normal and there was no history of HHT. Our findings would suggest that sEng acts differently in the presence of membrane-bound endoglin, compared with cases in which membrane-bound endoglin is reduced. Interestingly, one of the hallmarks of HHT1 is the deficient expression of endoglin (Abdalla and Letarte, 2006; Ruiz-Llorente et al., 2017) and, accordingly, it could be speculated that addition of exogenous sEng in this context could have a beneficial effect in counteracting the formation of AVMs. However, the underlying mechanism of action of sEng in AVM formation in both sporadic cases and in HHT remains to be elucidated.

In addition to the putative role of sEng in the TGF-β/BMP9 pathway, its function as a modulator of cell adhesion cannot be excluded. In this regard, the extracellular region of endoglin displays, within its zona pellucida domain, an RGD motif, which is a consensus sequence implicated in integrin-based interactions with other proteins (Gougos and Letarte, 1990; Saito et al., 2017; Llorca et al., 2007). Accordingly, it has been shown that sEng can modulate integrin-mediated cell adhesion involving membrane-bound endothelial endoglin (Rossi et al., 2013, 2016; Ruiz-Remolina et al., 2017), and this function might have an impact on the active angiogenesis and vascular remodeling processes in the neovascularized retina of *Eng*-iKO^e^ mice. In this regard, upon an inflammatory stimulus, leukocyte recruitment to the vasculature involves endothelial endoglin, via leukocyte integrins (Rossi et al., 2013), as well as BMP9 (Mitrofan et al., 2017). Because the inflammatory infiltrate of leukocytes appears to be involved in the vascular remodeling leading to AVMs in HHT patients (van Laake et al., 2006; Zhang et al., 2016), a role for sEng in this cell adhesion-dependent process can be postulated (Dingenouts et al., 2015; Rossi et al., 2015).

Endoglin and ALK1 play a key role in angiogenesis (López-Novoa and Bernabeu, 2010; Núñez-Gómez et al., 2017; Mahmoud et al., 2011; Roman and Hinck, 2017), and targeting these proteins has been used as a therapeutic approach to treat tumor angiogenesis. For example, TRC105 is a humanized monoclonal antibody against endoglin that not only binds to membrane-bound endoglin, but is also able to stimulate shedding of sEng (Kumar et al., 2014; Rosen et al., 2014). TRC105 is currently used in antiangiogenic therapies of several types of cancer (Liu et al., 2014; Paauwe et al., 2016). Moreover, ongoing clinical trials are testing two ALK1-related pharmacological inhibitors: dalantercept/ACE-041, an ALK1 extracellular domain fusion protein, and PF-03446962, an antibody against the extracellular domain of ALK1 (de Vinuesa et al., 2016). Whether sEng can also be used as an antiangiogenic drug remains to be determined.

Taken together, our results suggest that sEng has context-dependent effects. In the presence of endogenous transmembrane endoglin, it has antiangiogenic properties, but, in the absence of endogenous endoglin, sEng can rescue angiogenic defects in HHT1. This would suggest that under these latter conditions, sEng is pro-angiogenic. However, this hypothesis needs to be confirmed by further investigations on the exact mechanistic role of sEng in the absence of endogenous endoglin. Although we have shown that sEng can reduce the generation of AVMs, additional studies are needed to explore whether sEng is able to regress AVMs once they have been formed, and whether sEng could be used in the future as a therapeutic drug for the resolution of AVMs in HHT1 patients. These studies could also be helpful to better understand the function of sEng in conditions such as pre-eclampsia, cancer or inflammatory disease, where sEng is present at high levels.

## MATERIALS AND METHODS

### Cell culture and treatments

All cells were incubated routinely at 37°C in a humidified atmosphere with 5% CO_2_. HUVECs were purchased from Lonza and used at early passages (3-5). HUVECs were grown on 0.2% gelatin (Sigma-Aldrich) pre-coated plates, in endothelial growth medium (EGM-2) supplemented with 10% heat-inactivated fetal bovine serum (FBS, Gibco), 2 mM L-glutamine, 100 U/ml penicillin and 100 µg/ml streptomycin (Gibco), unless otherwise noted. MLECs were derived from *Eng^fl/fl^;RosaCre^ERT^* mice, as previously described (Anderberg et al., 2013). Briefly, endothelial cells were isolated with anti-CD31 (PECAM1)-coated Dynabeads sheep anti-rat IgG (Invitrogen) after dissociation of the lung tissue with 1 mg/ml collagenase, and cultured in MV2 endothelial cell basal medium (PromoCell) with 10% FBS. Treatment with 1 µM 4-OH-tamoxifen for 48 h was used to activate Cre^ERT^, leading to endoglin deletion. Treatments with human recombinant sEng (R&D Systems), reconstituted in PBS with 0.1% bovine serum albumin (BSA), were carried out either in serum-free medium or in 2% FBS medium, as indicated. For angiogenic protein assays, HUVECs or MLECs (with or without endoglin KO) were incubated with or without 100 ng/ml sEng for 24 h in EBM-2 or MV2 serum-free medium, respectively. The resulting culture supernatants were used to evaluate secreted angiogenic proteins using protein antibody arrays (described below). Culture supernatants were also analyzed using ELISA kits specific for mouse CXCL1 (DY453-05), coagulation factor III (DY3178-05), HB-EGF (DY8239-05) and MCP-1 (DY479-05) from R&D Systems. The immunoassays were performed according to the manufacturer's protocol and measured in a Glomax^®^ Multi Detection System (Promega).

### Wound healing and tube formation assays

*In vitro* scratch wounds were created by scraping confluent HUVEC monolayers in 24-well plates with a sterile pipette tip. Fresh EBM2 medium supplemented with 2% FBS and EGM2 (Lonza) with different concentrations of sEng was added, and samples were incubated for up to 10 h. Endothelial cell migration into the denuded area was monitored at 0, 4, 6, 8 and 10 h postwound. The ImageJ program (Schneider et al., 2012) was used to quantify the wound healing process. For tube formation assays, HUVECs were seeded on 24-well plates, previously covered with 100 μl standard Matrigel (BD Bioscience) diluted 1:2 in serum-free EBM2 medium supplemented with VEGF-containing EGM2 (Lonza). Samples were incubated at 37°C in EBM2 medium supplemented with 2% FBS and EGM2 in the presence of sEng (0-100 ng/ml) for 6 h, as indicated. Images were taken with an Olympus digital camera, and quantification of closed tubules was performed using Adobe Photoshop CS3 software.

### Angiogenesis protein arrays

Conditioned medium from HUVECs and MLECs cells, previously incubated with 100 ng/ml sEng in serum-free medium for 24 h, were used. The relative expression profile of angiogenesis-related proteins were quantified using a proteome profiler mouse angiogenesis array kit (ARY 015-mouse; ARY 007-human; R&D Systems) according to the manufacturer's instructions. The relative intensities of the spots were normalized per total protein concentration of each supernatant, measured with the BCA protein assay kit (Pierce), and by subtracting the background of the membranes. Each protein array experiment was performed in triplicate.

### Mice and treatments

The *Eng^fl/fl^* mouse and *Eng*-iKO^e^ mouse lines have been previously described (Mahmoud et al., 2010; Allinson et al., 2007). Neonates were injected subcutaneously with 0.5 mg tamoxifen (Sigma-Aldrich) at postnatal day (P) 2 and P4 to activate Cdh5-Cre*^ERT2^*, as reported (Mahmoud et al., 2010). Controls were tamoxifen-treated *Eng^fl/fl^* littermates. For intraocular treatments, P5 neonates were anesthetized by 3% isofluorane and intraocularly injected with 0.3 µl of 100 ng/ml sEng in PBS/0.1% BSA or control solution (PBS/0.1% BSA) in the left and right eye, respectively, using a Hamilton syringe (80001 10 μl SYR) attached to a fine needle (Needle PRE-33013 TSK Laboratory).

### Whole-mount, immunofluorescence staining and analysis of different parameters in mouse retinas

Mouse eyes were enucleated immediately postmortem at P7. Retinas were prepared and stained following the whole-mount immunofluorescence protocol from Tual-Chalot et al. (2013). Briefly, retinas were fixed in 4% (wt/vol) paraformaldehyde in PBS and then stained with Alexa Fluor 488-conjugated *G. simplicifolia* isolectin B4 (Life Technologies). Immunostaining using antibody against mouse endoglin (MJ7/18, eBioscience) was detected using an Alexa Fluor 568-conjugated secondary anti-rat antibody (Thermo Fisher Scientific). Vascular smooth muscle was detected using anti-αSMA-Cy3 antibody (Sigma-Aldrich). Stained retinas were flat mounted using ProLong Gold Antifade Mount medium (Thermo Fisher Scientific) and examined under a Zeiss Axioimager microscope, and images were analyzed using Zeiss-ZEN software. Analysis of vascular parameters was performed using Fiji-ImageJ2 software (Schindelin et al., 2012). Vascular migration was calculated by dividing the length of the vessel radius by the total length of the retina radius, both measured from the center of the retina in at least three different directions. Analysis of the branch points was calculated by counting the closed capillaries in at least three different areas near the retinal periphery. Measurements of the number of filopodia, at the leading edge of migrating tip cells, were normalized to the selected field of view, in at least four different sections of the retina.

The percentage area covered by isolectin-positive ECs, and αSMA- or endoglin-positive cells was calculated as the area occupied by the intensity of each marker normalized by the total retinal vascular area. Nonspecific staining at the edges of the retina or remnants of the hyaloid vessel were discarded. The number of AVMs was measured throughout the retina. At least 15 retinas were analyzed per parameter and per group.

### Statistical analysis

Wound healing, tubulogenesis and array data were analyzed by Student's *t*-test. ANOVA test was used for the comparison of more than one group. Nonparametric data were analyzed using the Kruskal–Wallis test. Variances between the different groups were determined by the Levene's test. Post hoc Tamhane or Bonferroni tests were performed for homogeneous or nonhomogeneous variances, respectively. Analysis was performed using SPSS software. All results shown in graphical representations are the mean of a minimum of three replicates. Data are presented as mean±s.e.m. with the corresponding *P*-values.

### Ethics

All animal experiments were performed under UK Home Office license, with approval from the Newcastle University Ethical Review Committee and following the guidelines of EU Directive 2010/63/UE for animal experiments. All applicable international, national and/or institutional guidelines for the care and use of animals were followed.

## Supplementary Material

Supplementary information

First Person interview
