# Supplementary material for: Soluble endoglin regulates expression of angiogenesis-related proteins and induction of arteriovenous malformations in a mouse model of hereditary hemorrhagic telangiectasia
Source: Dis Model Mech. 2018 Sep 21;11(9):dmm034397. doi: 10.1242/dmm.034397 (PMC6176985; doi:10.1242/dmm.034397)
Supplement: Supplementary information [file dmm-11-034397-s1.pdf]

© 2018. Published by The Company of Biologists Ltd | Disease Models & Mechanisms (2018): doi:10.1242/dmm.034397: Supplementary information

FIRST PERSON

First person – Eunate Gallardo-Vara

First Person is a series of interviews with the first authors of a selection of papers published in Disease Models & Mechanisms, helping early-career researchers promote themselves alongside their papers. Eunate Gallardo-Vara is first author on 'Soluble endoglin regulates expression of angiogenesis-related proteins and induction of arteriovenous malformations in a mouse model of hereditary hemorrhagic telangiectasia', published in DMM. Eunate is a postdoctoral researcher in the lab of Carmelo Bernabéu at Consejo Superior de Investigaciones Científicas (CSIC), Madrid, Spain, investigating signaling pathways (such as the TGF-β pathway) related to vascular diseases and benign tumors. She conducted the work in this article on two collaborative visits to Helen Arthur's lab at the Centre for Life, Newcastle, UK, as part of her PhD project in the Bernabéu lab.

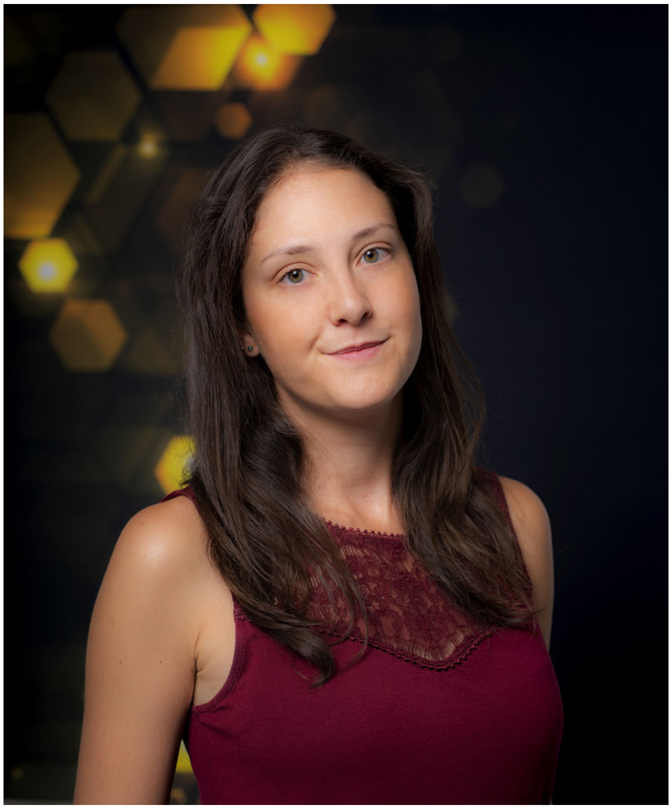

Eunate Gallardo-Vara

How would you explain the main findings of your paper to non-scientific family and friends?

Hereditary hemorrhagic telangiectasia (HHT) is a rare vascular disease, causing lesions in the blood vessels, that occurs in about 1:5000 people. HHT is a genetic autosomal dominant disease, meaning that each family where one of the parents is affected has a 50% chance of having a child with this condition. Unfortunately, current treatments for HHT remain just at the palliative level. My work is focused on HHT1 (one type of HHT), where the gene called endoglin is mutated. This gene encodes a cell membrane receptor protein, predominantly found in endothelial cells (the cells that line the inside of blood vessels). Endoglin plays a role in the conformation of normal vessels and is considered to promote the growth of new blood vessels. For example, endoglin production is increased in tumoral vessels, where it also plays an important role. In HHT1, the decrease in endoglin production affects blood vessel integrity, capillaries become more fragile and may disappear, leading to arteriovenous malformations in different organs such as the lungs, brain and gastrointestinal tract.

Soluble endoglin protein is produced by shedding of membrane-bound endoglin through the action of another protein, metalloprotease MMP14. Previous reports have shown that soluble endoglin inhibits the growth of new blood vessels, but nothing is known about its activity in an HHT1 background. In this paper, we wanted to get deeper into the function of soluble endoglin in an HHT1 mouse model. Why in an HHT mouse model? Because there is no current treatment for this disease, discoveries in this model may be useful in the future to improve patient wellbeing, and also to better understand the function of affected protein signaling pathways. As some therapies for HHT have already been described that involve inhibiting the growth of new blood vessels, we wondered if soluble endoglin was able to modulate the formation of the vascular lesions.

What are the potential implications of these results for your field of research?

The results of this work have increased and deepened our knowledge of the action and function of soluble endoglin, either in the presence or absence of membrane-bound endoglin. These results help us to better understand not only how this protein modulates the signaling of angiogenic pathways under normal conditions and in HHT disease, but also in tumoral vessels and other diseases where pathological angiogenesis presents together with soluble endoglin in the extracellular media. Our experiments into the intraocular local treatment of retinas with soluble endoglin, in which we were able to see a reduction of vascular lesions, introduce a potential future local therapy. Moreover, knowledge of this intraocular treatment could be a useful and rapid method to test new potential therapies for other vascular diseases.

What are the main advantages and drawbacks of the model system you have used as it relates to the disease you are investigating?

Our inducible endoglin knockout endothelial murine model provides the opportunity to induce deletion of the endoglin gene at the desired developmental stage. This is crucial because the mutation in homocigosis is embryonic lethal at postnatal day 10-12. The retina is the best model to see how the vasculature is formed as the neoangiogenic process takes place in postnatal days 0 to 8. By using this method, it is not necessary to wait until the adult stage. This is a great advantage in order to get faster results. It also gives us the opportunity to test different drugs in a localized manner and make an analysis of how the vasculature is affected almost immediately. One additional advantage of this model is that endoglin silencing occurs only in endothelial cells, which allows us to study the impact of

Eunate Gallardo-Vara's contact details: Centro de Investigaciones Biológicas, Consejo Superior de Investigaciones Científicas (CSIC), Madrid 28040, Spain.
E-mail: eunategv@cib.csic.es

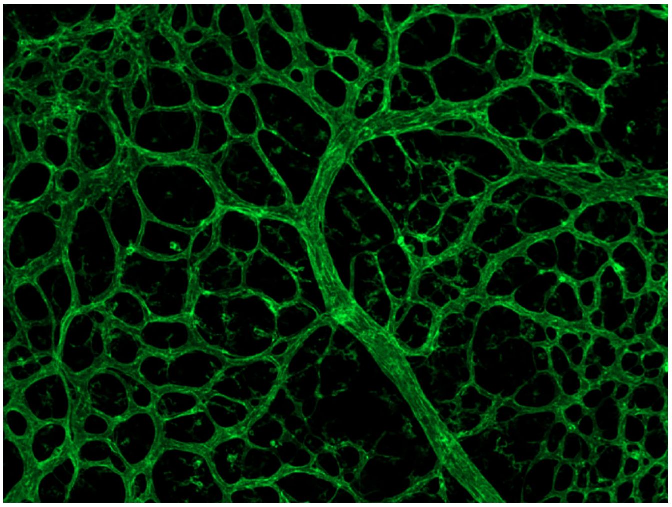

Arteriovenous malformations in endoglin knockout mouse retina cells stained with isolectin.

treatments specifically on this cell type. By contrast, a disadvantage of our mouse model is that endoglin suppression does not occur in other cells, such as pericytes or fibroblasts, which are present in the vasculature and also express endoglin.

From my point of view, animal models are crucial for any translational research, since they are the transition between the cell model and humans. Their use is fundamental to understanding the processes of diseases such as, in our case, angiogenesis and the implications of soluble endoglin in the retina neovessel network development.

"Our inducible endoglin knockout endothelial murine model provides the opportunity to induce deletion of the endoglin gene at the desired developmental stage."

What has surprised you the most while conducting your research?

We really didn't know what would happen with soluble endoglin treatment. It was a surprise to find out that it may, to some extent, mimic the action of the membrane endoglin that was not expressed in our mouse model.

Describe what you think is the most significant challenge impacting your research at this time and how will this be addressed over the next 10 years?

In the field of vascular diseases, the most significant challenge is understanding all the mechanisms that give rise to vessel formation, not only in pathological conditions, but also in physiological ones, and in a context-dependent manner. I believe that in the next ten years, new methods and technologies, such as new and improved animal models, and also *in vitro* 3D cell-derived organ models, will help to complete our understanding of why endothelial and other cell types behave differently depending on their position and extracellular and/or cellular environment. This will have important clinical implications. Currently HHT is a hereditary disease with no cure, so the most significant challenge will be to find a cure to increase the quality of patients' lives. In that sense, since it shares common signaling pathways with other diseases like cancer and benign hemangiomas, finding a treatment for HHT will also potentially open doors to therapies for other vascular diseases.

What changes do you think could improve the professional lives of early-career scientists?

"Research in the lab is hard work, and so sharing results is an important task."

We need to have more opportunities in our early careers as scientists: more fellowships to allow adequate pre- and postdoctoral training, and afterwards, the opportunity to learn the special knowledge of a specific field and the time to gain experience, which will allow the possibility to build a more stable scientific career. Of course, equal opportunities for women and men in all aspects of their scientific careers are really important, as personal life must be considered part of the scientist's life. Improving communication between scientists from different groups and specialties could help us to be more efficient and faster in different areas of research. Research in the lab is hard work, and so sharing results is an important task. I think that any knowledge or discovery, however apparently small, should not fall on deaf ears as it may further our understanding of the molecular and cellular basis of a disease.

What's next for you?

My dream is to contribute to scientific knowledge in the area of vascular research. Thus, I am applying for postdoctoral positions and fellowships to improve my knowledge in the cardiovascular and cancer fields. I would especially like to be engaged in translational research that advances molecular and cellular understanding of signaling pathways.

Reference

Gallardo-Vara, E., Tual-Chalot, S., Botella, L. M., Arthur, H. M. and Bernabeu, C. (2018). Soluble endoglin regulates expression of angiogenesis-related proteins and induction of arteriovenous malformations in a mouse model of hereditary hemorrhagic telangiectasia. *Dis. Model. Mech.* **11**, dmm034397.
